# The Dominant Role of Visual Motion Cues in Bumblebee Flight Control Revealed Through Virtual Reality

**DOI:** 10.3389/fphys.2018.01038

**Published:** 2018-07-31

**Authors:** Elisa Frasnelli, Natalie Hempel de Ibarra, Finlay J. Stewart

**Affiliations:** ^1^Department of Evolutionary Studies of Biosystems, The Graduate University for Advanced Studies, Hayama, Japan; ^2^School of Life Sciences, University of Lincoln, Lincoln, United Kingdom; ^3^Department of Psychology, College of Life and Environmental Sciences, University of Exeter, Exeter, United Kingdom

**Keywords:** optic flow, vision, motion, flight, virtual reality, closed-loop, free flight, bee

## Abstract

Flying bees make extensive use of optic flow: the apparent motion in the visual scene generated by their own movement. Much of what is known about bees' visually-guided flight comes from experiments employing real physical objects, which constrains the types of cues that can be presented. Here we implement a virtual reality system allowing us to create the visual illusion of objects in 3D space. We trained bumblebees, *Bombus ignitus*, to feed from a static target displayed on the floor of a flight arena, and then observed their responses to various interposing virtual objects. When a virtual floor was presented above the physical floor, bees were reluctant to descend through it, indicating that they perceived the virtual floor as a real surface. To reach a target at ground level, they flew through a hole in a virtual surface above the ground, and around an elevated virtual platform, despite receiving no reward for avoiding the virtual obstacles. These behaviors persisted even when the target was made (unrealistically) visible through the obstructing object. Finally, we challenged the bees with physically impossible ambiguous stimuli, which give conflicting motion and occlusion cues. In such cases, they behaved in accordance with the motion information, seemingly ignoring occlusion.

## Introduction

Like many other animals, foraging bees would literally be lost without their sense of vision; they use visual information to navigate, stabilize their flight, avoid collisions, and execute smooth landings (Serres et al., 2008; Baird et al., 2013; Portelli et al., 2017; also recently reviewed by Altshuler and Srinivasan, 2018). To perform these behaviors successfully, they must infer the 3D structure of the world from the 2D images formed on their retinas. Bees and other insects achieve this using optic flow: the wide-field pattern of visual motion created by one's own movement (Gibson, 1950; Lehrer, 1991; Krapp and Hengstenberg, 1996; Kral, 1998; Linander et al., 2015). In particular, visual motion can be used to judge the proximity of visual objects: for a given speed of translational flight, the speed at which an object appears to move in the visual field is inversely proportional to its distance.

Bees exhibit a high degree of maneuverability, i.e., the ability to precisely control their flight and thus avoid collisions (Crall et al., 2015). This behavior is largely guided by visual motion cues. Honeybees (*Apis mellifera* L.) regulate their flight speed by maintaining a constant speed of visual motion (Srinivasan et al., 1996), using the lateral, ventral, as well as dorsal parts of their visual field (Portelli et al., 2011). However, optic flow in the ventral visual field seems to become more important in open environments; bumblebees (*Bombus terrestris*) trained to fly along a tunnel rely more on motion cues from the ground as the tunnel width increases (Linander et al., 2016). Furthermore, honeybees also use visual information from the ventral visual field to control their altitude (Baird et al., 2006; Portelli et al., 2010). Both honeybees and bumblebees also seem to actively maximize the extraction of visual motion information by performing pivoting flight maneuvers when approaching and leaving salient locations. These maneuvers serve to stabilize their gaze and maximize the motion parallax information available (Boeddeker and Hemmi, 2010; Riabinina et al., 2014).

In addition to wide-field motion cues, honeybees can use discontinuities in the optic flow field to identify and target objects, even if the objects are camouflaged, i.e., bearing a similar texture to their background (Zhang et al., 1995). The greater the difference in distance of the object and background, and thus the greater the disparity in speed of visual motion, the better honeybees are able to detect the raised object (Srinivasan et al., 1990). When faced with a camouflaged raised platform, honeybees tend to land on its edges while facing inwards, consistent with motion discontinuities being a salient perceptual feature (Lehrer and Srinivasan, 1993; Josef, 2018). In the converse case of a camouflaged hole though which a more distant surface is visible, honeybees approach the center of the hole, avoiding the edges (Baird et al., 2013). This indicates that the polarity of motion edges plays an important role in guiding flight behavior. There is evidence that honeybees can gauge the degree of motion discontinuity to quantitatively estimate object distance: they can be trained to select artificial flowers of variable size at a specific depth below a Perspex sheet based on motion cues (Lehrer et al., 1988; Srinivasan et al., 1989).

The aforementioned studies convincingly demonstrate the importance of motion cues for bee flight. However, bees are often extensively trained in such studies, and as such, one may question the ecological validity of the experimental paradigm. For instance, in the case of the camouflaged hole, honeybees were trained to land *on* the transparent plastic covering the hole (Srinivasan et al., 1990; Lehrer and Srinivasan, 1993). Foraging from a transparent surface is presumably not a behavior that bees would exhibit under natural conditions, yet their impressively flexible learning capabilities allow them to complete the task given sufficient training (at least 30 rewarded visits in this case). Furthermore, these studies' use of physical objects makes it difficult to exclude the possibility that other visual cues besides motion (e.g., stereopsis, occlusion, or texture)—or indeed other sensory modalities entirely—may also contribute to the behaviors observed.

Modern virtual reality (VR) techniques provide unprecedented opportunities to test freely behaving animals in complex visual environments, in which cues can be manipulated independently, potentially in ways that would be impossible to achieve in traditional experiments (Fry et al., 2008; Dombeck and Reiser, 2012; Peckmezian and Taylor, 2015; Stowers et al., 2017). Because virtual environments are not constrained by the laws of physics, objects can for instance appear and disappear arbitrarily, or even be at apparent distances beyond infinity (Schuster et al., 2002). A further benefit of VR is that it places fewer constraints on the animals' responses; they can potentially move through virtual surfaces, unlike those made of Perspex or cardboard. In the present study, we used a closed-loop tracking and display system to modulate the visual stimulus in real time as a function of the animal's movements. In this way, we created virtual objects that appeared to be situated in 3D space outside the monitor surface. Similar approaches have been previously used to study visually-guided behavior in other insects such as locusts (Wallace, 1959), fruit flies (Schuster et al., 2002), and butterflies (Stewart et al., 2015), as well as rodents and fish (Stowers et al., 2017). This study is (to our knowledge) the first to employ VR techniques to investigate bees, animals noted within the neuroethology community for their agile flight and impressive capacity for “cognitive” tasks such as learning and navigation.

**Figure 1:**
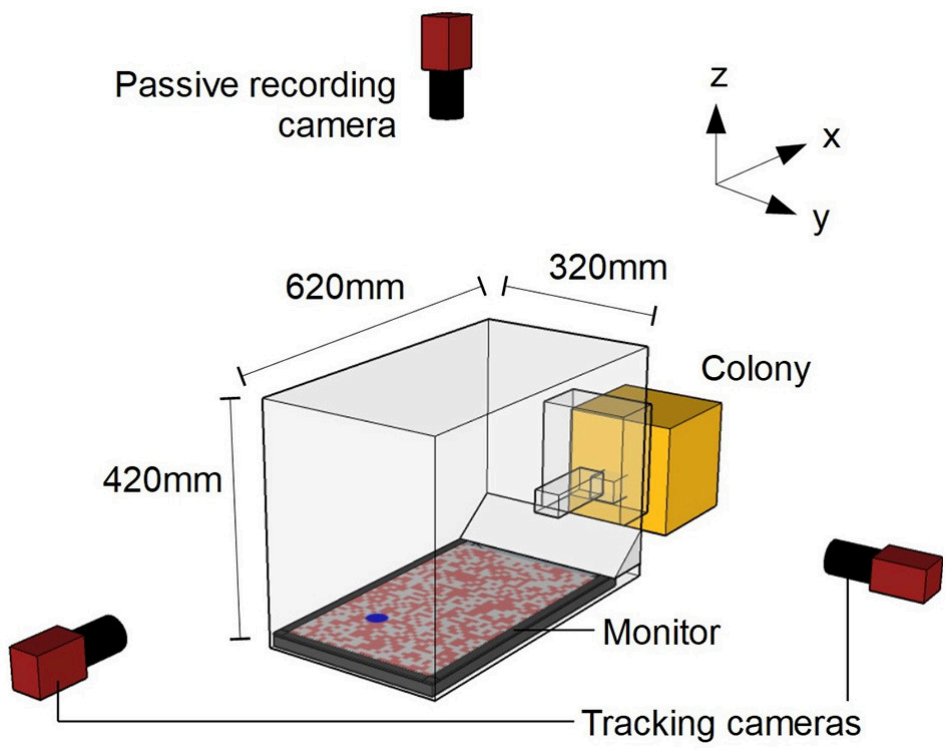
Schematic diagram of experimental setup. Camera positions are not to scale. The origin of the co-ordinate system is defined as the center of the monitor surface.

We briefly trained bumblebees to feed from a static colored target displayed on a monitor placed flat on the arena floor (Figure 1). Motivated foragers returned repeatedly to collect more food, and we could therefore present them with a sequence of unrewarded test trials in which they had to negotiate a virtual obstacle before reaching the target. These virtual obstacles in the tests were either “congruent,” i.e., faithfully simulating 3D arrangements of objects, or “incongruent,” i.e., where motion cues gave conflicting information to other visual cues such as occlusion. The latter case allowed us to test which cues dominate the bee's visuomotor responses to obstacles encountered during flight.

## Materials and methods

### Animals

Female worker bumblebees, *Bombus ignitus*, from two commercially reared colonies (Agrisect, Japan) were used in the experiments. In addition to the syrup that some individuals received during training and testing, every evening the whole colony was fed with 15 ml of syrup and approximately 1.5 g of pollen (Agrisect, Japan). For the whole duration of the experiments the bees were kept in the colony inside a laboratory room at the School of Advanced Sciences, Sokendai (The Graduate University for Advanced Studies) in Japan, where the experiments were carried out. The temperature in the room was maintained at 25 ± 2°C, with a 12:12 h light:dark cycle.

### Experimental set-up

The bee colony was placed into a custom-made set-up (Figure 1) that allowed the bees to emerge from the colony into a Plexiglas chamber (15 × 10 × 15 cm; l × w × h). The chamber was connected to an experimental arena (62 × 32 × 42 cm) through a Plexiglass corridor (24.5 × 4.5 × 4.5 cm). The corridor had two vertical sliding doors that allowed the experimenter to selectively release marked individuals into the arena (Supplementary Video 1). The arena walls and ceiling consisted of a frame of black aluminum bars covered with plastic white netting of 2 × 5 mm rectangular holes. The arena floor was a 24″ (61 cm) LCD monitor (Benq XL2420T). Light emitted from LCD monitors is highly linearly polarized, potentially providing a directional reference. However, due to the twisted rhabdoms in their photoreceptors, bees are insensitive to polarization in the ventral visual field (Hempel de Ibarra et al., 2014). An untextured white cardboard ramp extended from one edge of the monitor to the underside of the entrance tunnel, to cover the gap between the monitor and wall. Two orthogonally oriented cameras (AVT Prosilica GE680, monochrome) were used to track individual bees during the experiment. These two cameras captured 100 frames s^−1^ with a 2.0 ms exposure at a resolution of 640 × 480 pixels (the camera oriented along the x-axis was placed in a portrait orientation to better match the dimensions of the arena). An additional camera (AVT Prosilica GE1050, monochrome) was placed above the arena in order to record the behavior of the insects during testing (50 frames s^−1^, 4.0 ms exposure at a resolution of 1,024 × 600 pixels).

### Experimental procedure

We displayed a blue circle (4 cm diameter, RGB: 0, 0, 255) in a random position on the monitor. The monitor displayed a pink (RGB: 255, 127, 127) and white (RGB: 255, 255, 255) random chequerboard pattern of 10 mm squares (Figure 1). (The red component of the display should be virtually invisible to bees, but provides a bright backdrop for the overhead video footage, allowing us to more easily detect leg extension (Hempel de Ibarra et al., 2014). We refer to the blue circle as the target and to the pink-and-white pattern as the background. The chequerboard pattern was necessary for the bees to stabilize their flight Linander et al., 2017. An Eppendorf tube cap mounted on a transparent acrylic sheet (60 × 80 mm) was filled with syrup and placed over the target.

Naive bees were individually released from the colony into the arena through the corridor and allowed to fly for 10 min inside the arena. No bee spontaneously fed from the feeder during this time. The bee was then carefully caught into a tube and marked with a colored number tag. After 5 min, the bee was manually placed onto the target and allowed to feed *ad libitum*. When bees finished feeding, we gave them 5 min to return to the colony by themselves. If they failed to do so, they were caught and placed back into the colony.

In all subsequent training sessions, the target appeared at least 160 mm from its previous location, to prevent the bee from learning its position. Training was repeated, at most two sessions per day (for a maximum of five sessions in total, although 90% of the bees required only two sessions) until the bee spontaneously fed within 10 min in two consecutive sessions.

During testing, the various conditions were presented in randomized order, with no reward. We performed training sessions between each test session to maintain the bee's foraging motivation. If a bee failed to approach the target for the whole duration of a test session, we repeated it. On a given day, each bee was tested repeatedly until it either stopped making foraging flights or successfully completed six unrewarded test conditions. This procedure was repeated on subsequent (potentially non-consecutive) days until the individual either completed all conditions or stopped emerging from the colony (presumably because it had died). Each bee completed each condition at most once; not all bees completed all conditions. For each condition, the number of individuals that successfully approached the target is reported as the *n*-value.

### Tracking

The real-time tracking procedure was similar to that described in Stewart et al. (2015), controlled by a custom-written Java program utilizing the ImageJ API. Prior to running experiments, the projection matrices of the two tracking cameras were calculated by manually identifying the positions of the arena vertices in the camera image, and then performing a least-squares fit. This represents a rectilinear approximation; radial distortion from the lenses was not accounted for. Immediately before commencing each experimental trial, a background image of the empty arena was obtained for each camera by taking the median of 19 consecutive video frames. Once the experiment was underway, each captured frame was subtracted from the saved background image. The resulting image was thresholded, and particle analysis was used to identify the largest connected region of supra-threshold pixels, corresponding to a dark object, i.e., the bee's body. The centroid of this region, together with the projection matrix of the camera, define a ray in 3D space along which the bee must lie. The mutually orthogonal line connecting the two cameras' rays at their closest point was calculated, and if this was <30 mm in length (i.e., the rays approximately intersected) then its midpoint was taken as the bee's 3D position. If its length exceeded this value, a tracking error was assumed to have occurred and that frame was ignored. The bee's trajectory, i.e., the series of timestamped 3D positions, was recorded. Additionally, timestamped images from the overhead camera were recorded for offline analysis.

### Virtual reality display

The “virtual” objects created in the tests were static two-dimensional shapes bearing a similar pink-and-white random chequerboard pattern to the background. They were located on a horizontal plane 60 mm above the surface of the monitor. The objects were an unbroken virtual floor, a virtual floor with a square hole, and a rectangular platform. As the hole and platform objects allow the background plane to be seen “behind” them, we refer to them as “foreground.” The projections of these objects onto the plane of the monitor from the bee's position were calculated in real-time as each display frame was generated, based on the last detected 3D position from the tracking system. Thus, while the virtual objects were static in 3D space, their position and size on the display changed as the bee flew. From its perspective, this created the illusion of depth via visual motion cues. The latency in the feedback loop was approximately 50 ms. When the bee descended to an altitude lower than that of a virtual object, the object would disappear from the screen, as it would no longer appear in the animal's ventral visual field. The stimulus presentation did not begin until the bee first ascended to an altitude of 350 mm after leaving the entrance tunnel (Supplementary Video 1). Prior to this, the screen displayed a uniform background of light pink (RGB: 255, 191, 191; midway between the two pattern colors).

### Test conditions

**Figure 2:**
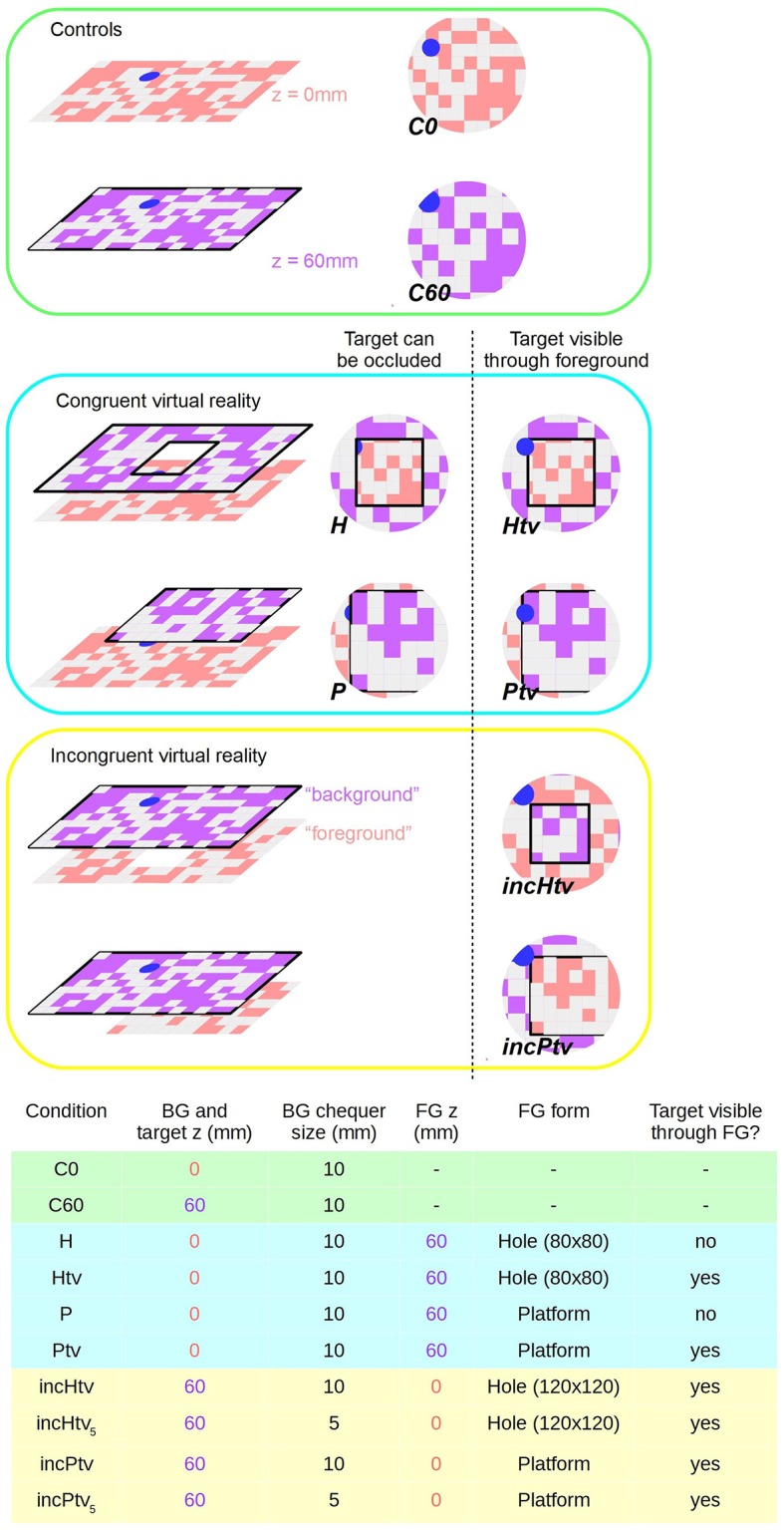
Virtual reality conditions. **Left:** schematic diagram showing surfaces at virtual altitudes of 0 mm (i.e., static, pink) and 60 mm (closed-loop, purple). Right: representation of bee's ventral visual field; the center of the circle is the nadir. Pink and purple coloring is to denote z-depth only; actual chequerboard patterns are all pink/white. Chequerboard texture is not to scale. **Bottom:** Table summarizing the parameters that differ between conditions. BG stands for background; FG for foreground. Shading indicates which group (control, congruent VR, incongruent VR) each condition belongs to. Conditions incHtv_5_ and incPtv_5_ are not shown, but are simply incHtv and incPtv respectively with the spatial frequency of the background (purple) texture doubled.

We can divide our test stimuli into three groups: controls (C0, C60), congruent (P, H, Ptv, Htv) and incongruent (incPtv, incHtv) (Figure 2; Supplementary Videos 2–9). These abbreviations are explained below.

In the control conditions, no foreground object was present, only the background and colored target:

- **C0** (control; background and target at *z* = 0 mm): We displayed the target and background statically on the monitor, as in the training condition but without reward (Supplementary Video 2). The position of the target was still subject to the constraints described for the congruent conditions below, despite the absence of an obstacle. *n* = 28.
- **C60** (control; background and target at *z* = 60 mm): The background and target are presented in closed-loop at a virtual altitude of 60 mm, without any occluding foreground objects (Supplementary Video 3). When the bee dropped below the background plane, the monitor displayed uniform pink, as before the start of the experiment. *n* = 24.

In the congruent conditions, the target and background were displayed at the monitor level (*z* = 0 mm). In addition, we displayed a virtual object as an obstacle at 60 mm above the monitor (foreground; *z* = 60 mm), as detailed below. The target's position was randomized for every test trial, subject to certain constraints: it would be completely under either foreground object (and thus hidden from directly overhead), but with its edge within 20 mm of the object's edge, giving the bee a chance to glimpse it from shallower elevations as it flew. The following tests were conducted:

- **H** (hole): We displayed a foreground obstacle covering the entire monitor, except for a square hole extending from (−40 mm, −40 mm) to (40 mm, 40 mm), with (Crall et al., 2015) being the center of the monitor (Supplementary Video 4). *n* = 25.
- **Htv** (hole, target always visible): Same as (H) except that the target was always visible, i.e., not occluded by the foreground (Supplementary Video 5). Thus, the bee could potentially approach the target directly, ignoring the obstacle. *n* = 26.
- **P** (platform): We displayed a raised rectangular platform extending from −100 to 100 mm in the y-dimension and from −100 mm to infinity (i.e., beyond the wall of the arena, on the side of the entrance tunnel) in the x-dimension. This asymmetry was introduced to prevent the animals approaching the target from the vicinity of the tunnel, because descents to this region might represent attempts to return to the colony rather than to land on the target (Supplementary Video 6). *n* = 26.
- **Ptv** (platform, target always visible): Similar to the (P) condition but with the target displayed at all times (Supplementary Video 7). *n* = 26.

In the physically impossible “incongruent” conditions (Supplementary Videos 8, 9), we manipulated the stimuli so that motion cues and occlusion cues were put in conflict. The “background” and target now appeared at *z* = 60 mm and the “foreground” at *z* = 0 mm, but with the foreground occluding the background despite being more distant. In all incongruent conditions the target was always visible, i.e., was never occluded by the “foreground.” The target was always located “under” the obstacle as before, but because of the larger hole (see below), this actually constrained the range of possible positions more tightly than in the congruent conditions. We assume that bumblebees perceive occlusion cues in a similar way to honeybees, which have been shown to identify partially occluded objects and even recognize illusory contours (Hateren et al., 1990; Srinivasan, 2010).

- **incHtv** (incongruent hole): The “foreground” object was static on the monitor (*z* = 0 mm), while the “background” pattern visible through the hole moved in closed-loop with a virtual altitude of 60 mm (Supplementary Video 8). The hole was larger than in the congruent condition, extending from (−60 mm, −60 mm) to (60 mm, 60 mm). *n* = 25.
- **incPtv** (incongruent platform): The platform was static (*z* = 0 mm) and occluded the moving background (*z* = 60 mm) (Supplementary Video 9). The platform dimensions were the same as in P/Ptv. *n* = 24.

Given that the background and foreground had a similar texture, one could argue that the background at *z* = 60 mm would appear closer not only due to motion cues but also the apparent size of the squares, i.e., perspective cues. Therefore, we repeated the incHtv and incPtv conditions with a background chequerboard of size 5 mm. In this situation, the squares of the background appear smaller as long as the bee is at an altitude of at least 120 mm. In this way, perspective cues are put in agreement with occlusion cues, but in conflict with visual motion cues. We refer to these conditions as **incHtv**_5_ and **incPtv**_5_. We tested only a subset of bees in these conditions: *n* = 16 in both cases.

### Data analyses

**Figure 3:**
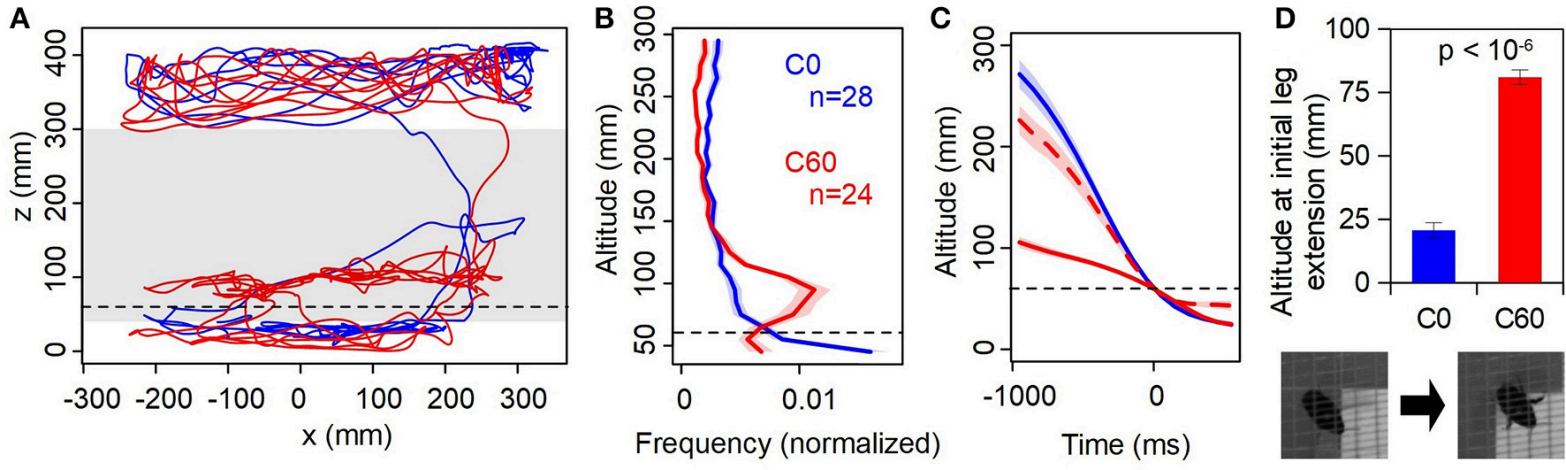
Physical vs. virtual floor. **(A)** Sample trajectories (1 min duration) from the same individual in the C0 (static, blue) and C60 (virtual reality closed-loop, red). Dashed line indicates the level of the virtual floor, gray shaded band is the range of altitudes shown in **(B)**. **(B)** Histogram of time spent at altitudes between 40 and 300 mm, averaged across all individuals. Shaded regions are ±1 SE. **(C)** Event-triggered mean altitude profiles; *t* = 0 is the first instance of the bee descending below *z* = 60 mm. Dashed red line is C60 data shifted downwards by 60 mm, i.e., expressed relative to the virtual as opposed to physical floor. **(D)** Mean altitude at which first leg extension is observed. Inset: stills from overhead camera showing a flying bee with legs retracted (left) and extended (right).

The automatically recorded 3D trajectories were smoothed using Gaussian averaging (s.d. = 50 ms) and resampled at 10 ms intervals. For each trial, we identified the first occasion where the bee descended through the plane of the virtual objects (z = 60 mm). Additionally, the first instance of the bee extending its legs, which is part of the insect's stereotyped landing response (Srinivasan et al., 2000; Tammero and Dickinson, 2002; Reber et al., 2016) was identified manually by observing the overhead video footage (Figure 3D, Supplementary Videos 2–9).

## Results

### Virtual floor perceived as a real surface

First, we compared the bees' approach and landing responses between two control conditions where there was no obstructing virtual object present in the foreground (Figure 2, C0 and C60; Supplementary Videos 2, 3). In the C0 condition the display is static, with both the background and target at altitude 0 mm. In C60 both the background and target are at an altitude of 60 mm, creating the illusion that the arena floor is above the monitor surface.

Figure 3A shows examples of flight trajectories from a single animal. In the C0 case (similar to the training condition), one can observe typical behavior where the bee flies near the ceiling of the cage before descending and hovering in the vicinity of the target prior to landing on it. Little time is spent at intermediate altitudes. When the background was at an apparent altitude of 60 mm above the physical ground (C60), the bee spent considerably more time hovering just above this level, before eventually “breaking through” the virtual background and landing on the monitor (which would be displaying uniform pink by this point). This tendency is clearly evident in the distribution of flight altitudes (Figure 3B). Bees in the C60 condition spent considerably more time at intermediate altitude (around 95 mm) compared to C0; the modal altitude over the range shown in Figure 3B is significantly higher in C60 than C0 (90~100 mm vs. 40~50 mm; *p* < 10^−5^, *n* = 24,28; Mann-Whitney test).

Bees descended much more slowly through the *z* = 60 mm plane in the C60 condition than in C0 (Figure 3C). However, measuring their altitude relative to the virtual floor rather than the monitor surface, the descent profile more closely resembles that seen in the C0 condition (dashed line in Figure 3C). The trajectory is slightly slower, which is likely because the descent from near the ceiling is shorter. Importantly, in the C60 condition bees extended their legs in preparation for landing 60 mm higher than in C0 (Figure 3D). Taken together, these observations indicate that the VR illusion created by our system is “convincing” to the bees, and that visual motion cues strongly influence their flight behavior.

### Avoidance of virtual obstacles

**Figure 4:**
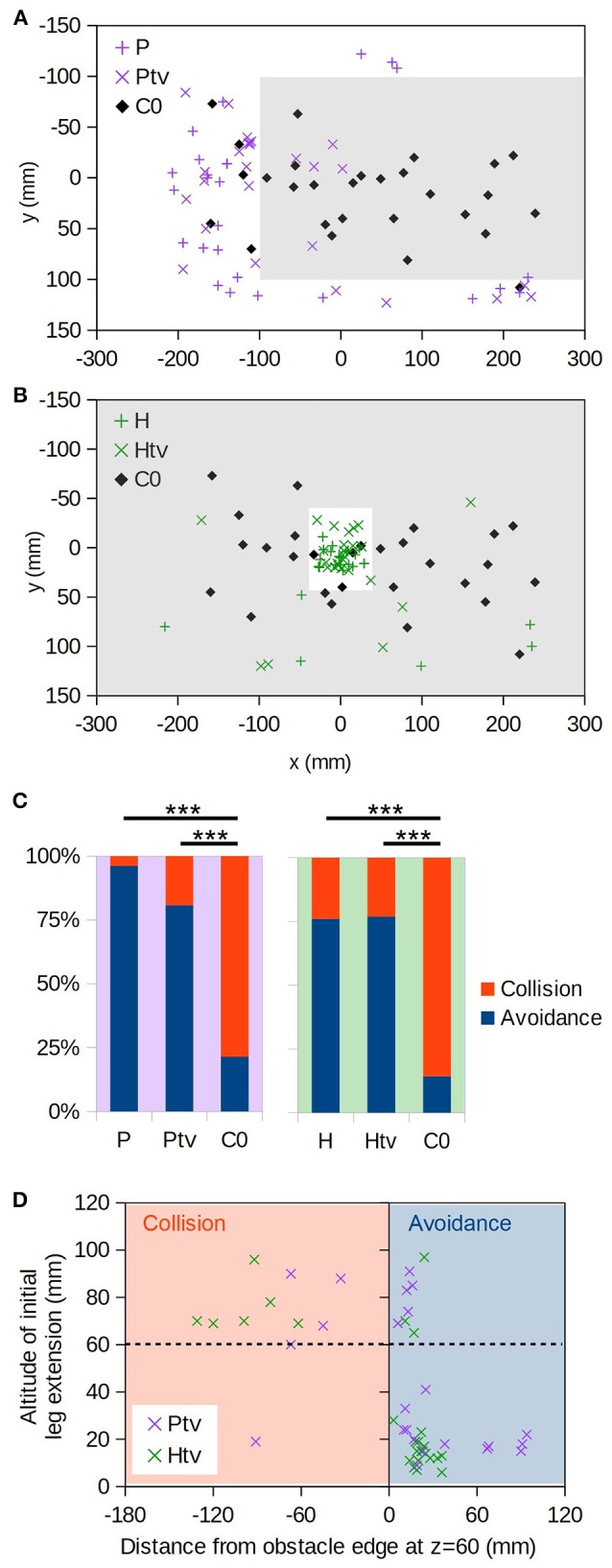
Virtual object avoidance. **(A)** (x,y) positions at which individuals initially descended through the plane of the virtual platform (i.e., *z* = 60 mm); the extent of the virtual platform is indicated by gray shading. Thus, descents occurring within the shaded area correspond to “collisions” with the virtual obstacle. Coloured “+” symbols are for the realistic occlusion condition (P), and “×” symbols for the condition where the target is always visible (Ptv). Black diamonds are the control condition, where no obstacle exists (C0). **(B)** As A, but for the hole obstacle. **(C)** Proportions of descents resulting in collision vs. avoidance. For C0 no virtual obstacle is present, but we assess whether each descent would have collided with the obstacle in question, thereby providing an estimate of the avoidance rate due to chance alone. “^***^” denotes *p* < 0.001 (Fisher's exact test); numbers of individuals are as follows: n_P_ = 26, n_Ptv_ = 26, n_H_ = 25, n_Htv_ = 26, n_C0_ = 28. **(D)** Altitude of initial leg extension as a function of distance from the edge of the virtual obstacle. Negative distances correspond to collisions (orange shading). Dashed line is the virtual obstacle plane.

We now turn our attention to cases where the bee must negotiate a virtual foreground obstacle positioned above the target: either a raised platform or a surface with a hole “cut” in it (Figure 2, H and P; Supplementary Videos 4, 6). Figures 4A,B (+symbols) show the (x,y) positions at which bees initially passed through the plane of the foreground object. They seldom broke through the virtual object, but instead descended around the platform (P) or through the hole (H).

It could simply be that the bees flew until they saw the target through the hole or around the platform, and then descended straight toward it, ignoring motion cues. To exclude this possibility, in the Ptv and Htv conditions the foreground object occluded the background but not the target. The target was thus visible at all times regardless of the bee's position with respect to the obstacle (Figure 2, Htv and Ptv; Supplementary Videos 5, 7). We found that the bees still avoided the foreground object in the majority of cases (Figures 4A,B; × symbols), and significantly more frequently than predicted by chance (Figure 4C; Ptv vs. C0: *p* < 10^−4^, *n* = 26,28; Htv vs. C0: *p* < 10^−5^, *n* = 25,28; Fisher's exact test). This strongly suggests that they were unable to ignore visual motion cues, even when given an unobstructed path to the target.

However, bees did occasionally fail to avoid the virtual obstacles, and broke through them. In such instances, they tended to extend their legs prior to apparent contact (Figure 4D, top-left quadrant). This implies that the animals still perceived the proximity of the virtual floor even if they did not avoid it. We also observed leg extension above 60 mm altitude in some descents that successfully avoided the obstacle, but these were all “near misses” where the animal came within 25 mm of the hole or platform edge (Figure 4D, top right).

### Motion cues override occlusion cues

**Figure 5:**
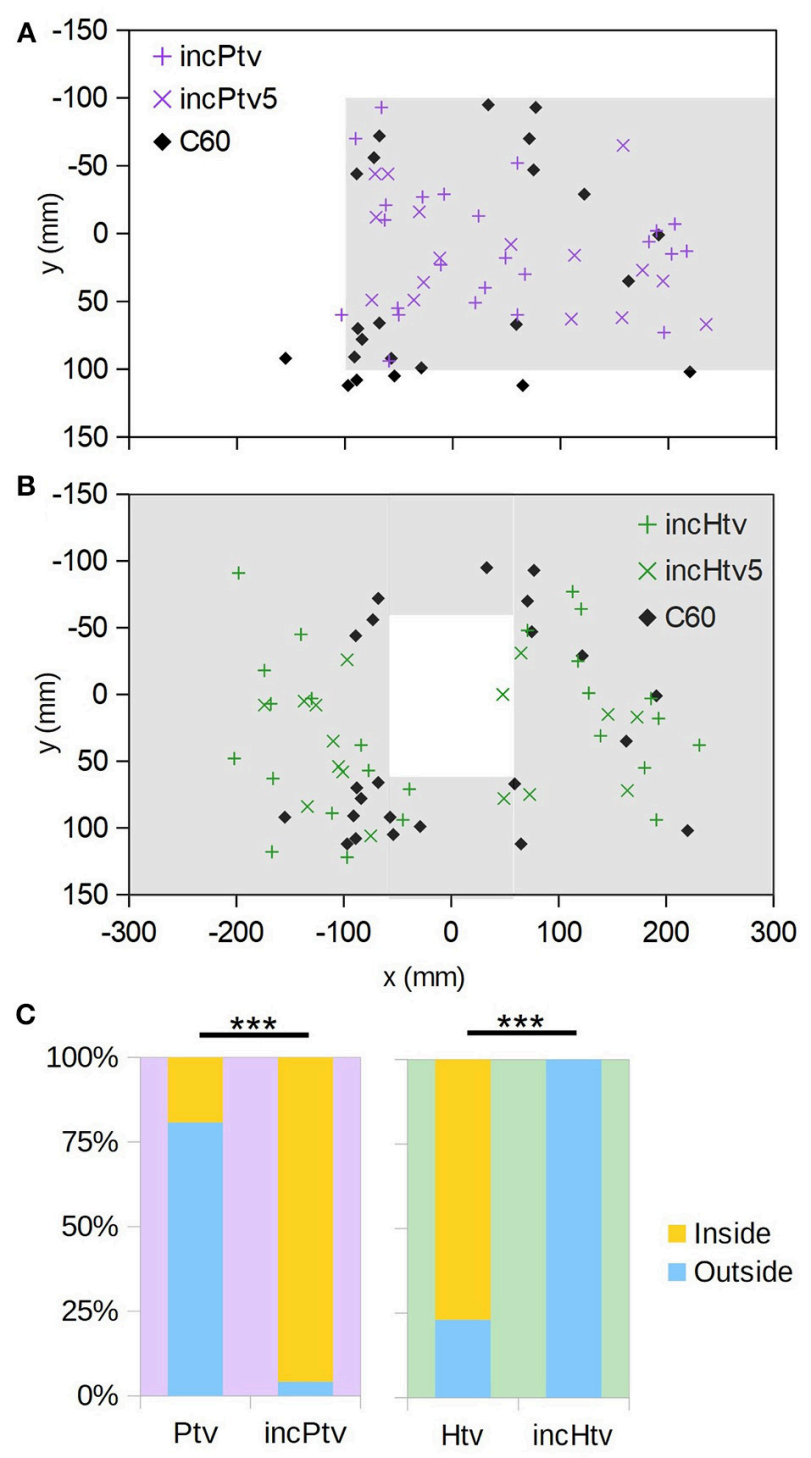
Incongruent virtual objects. **(A)** (x,y) positions at which individuals initially descended through the plane of the virtual “background” (i.e., *z* = 60 mm); the extent of the platform (*z* = 0 mm, but occluding the background) is indicated by gray shading. Coloured “+” symbols are for the case where the background chequerboard is composed of squares of the usual size (10 mm), “×” symbols for the case where they are 5 mm, and black diamonds are the control condition where no obstacle exists (C60). **(B)** As A, but for the incongruent hole obstacle. **(C)** Comparison with the congruent conditions (Figure 4) showing how frequently animals descended through the *z* = 60 mm plane inside vs. outside of the platform/hole. “^***^” denotes *p* < 0.001 (Fisher's exact test); numbers of individuals are as follows: n_Ptv_ = 26, n_incPtv_ = 24, n_Htv_ = 26, n_incHtv_ = 25.

To investigate the interplay between occlusion and motion cues, we displayed physically impossible “incongruent” stimuli where distant objects (based on motion cues) occlude proximal ones (Figure 2, incHtv and incPtv; Supplementary Videos 8, 9). In these conditions, the “background” and target were at an altitude of 60 mm, and the “foreground” at 0 mm. If bees follow occlusion cues, we would expect them to descend through the hole and around the platform as before. However, if motion cues dominate their flight behavior, they should perceive the regions inside the hole and *out*side the platform as being raised. Indeed, we observed that the bees typically flew around the incongruent hole, and through the incongruent platform (Figures 5A,B; + symbols). This behavior was not significantly different to that elicited in the C60 control, implying that they did not perceive the “foreground” as an obstacle to avoid. Compared to the congruent VR conditions (Figures 4A,B), however, we observed a clear inversion of their behavior (Figure 5C). Thus, it appears that the bees perceived the incongruent hole and platform as a platform and a hole, respectively, indicating that motion cues take precedence over occlusion cues in guiding their flight behavior.

It could be that in this ambiguous situation, the bees judged the distance of surfaces not on the basis of visual motion, but rather the apparent spatial frequency of the texture. To exclude this possibility, we doubled the spatial frequency of the “background” (*z* = 60 mm) pattern (incHtv_5_/incPtv_5_, Figures 5A,B; diamonds). Under these conditions, the background squares subtend smaller visual angles than those of the foreground pattern when the bee is over 120 mm above the arena floor. The behavior remained unchanged, consistent with motion cues being the primary mechanism by which proximity is gauged.

## Discussion

We briefly trained bees to feed from a randomly-positioned high contrast blue target which should be readily detectable from any position in the arena (Dyer et al., 2008; Wertlen et al., 2008). We then used a closed-loop tracking system to present virtual objects to the bees when they returned to forage in the arena. In all tests, bees responded to the virtual objects as if they were real obstacles, flying around them and/or extending their legs in anticipation of contact with them. These behaviors emerged spontaneously, in that the insects never experienced any punishment or reward when colliding with or avoiding the virtual objects. Even when the bees could unrealistically see the target through the obstacles (as though they possessed “X-ray vision”), they nevertheless avoided them. Our results confirm previous findings that motion cues are innate and dominant in the insect's flight control mechanisms (locusts and mantis: e.g., Kral and Poteser, 1997; honeybees: e.g., Srinivasan et al., 1989; fruit flies, e.g., Fry et al., 2009; van Breugel and Dickinson, 2012; butterflies: Stewart et al., 2015).

A key benefit of the VR technique is that it allowed us to generate physically impossible testing conditions, where various visual depth cues were put into conflict. For instance, one surface could occlude another and have a texture of a greater spatial period (implying that it is more proximal) but move more slowly in the visual field (implying that it is more distant). We found that in these ambiguous incongruent situations, the bees' flight was guided by motion cues whilst occlusion cues were ignored. This reflects Lehrer and Srinivasan's (1993) finding that bees preferentially approach a motion discontinuity (i.e., the visual border where one object occludes another) from the side appearing to move more slowly, i.e., the side of the more distant surface. However, our results indicate that the movement of the border itself does not need to match the movement of the more proximal surface, as it would in the case of static physical objects. This further reinforces our conclusion that visual motion is the dominant means by which bees estimate the spatial arrangement of nearby objects.

The illusion created by our system is not perfect. One limitation is that we tracked the body centroid of the bee rather than the head, which inevitably introduces an error on the order of 10 mm. While real-time automatic head tracking has been implemented for walking flies (Bath et al., 2014) and rodents (Stowers et al., 2017), this remains technically challenging for an insect freely flying in a large volume. Other factors adding noise to our estimate of the animal's position at any given moment include: imperfect calibration, lens distortion, sporadic tracking errors (caused by e.g., shadows or reflections), and latency (see Methods). Because of this noise, virtual objects presumably appear to jitter rather than maintain a perfectly stable position from the bee's perspective. One might argue that the bees are simply repelled by this jitter, rather than avoiding the virtual objects because of their perceived proximity. While we cannot completely exclude this possibility, we find this explanation implausible because the bees extended their legs as if to land when getting within a few centimeters of the virtual objects (Figures 3D, 4D), implying that they perceive them as existing at a specific spatial position.

Considering the natural situation in an outdoor environment, the bee's flight control system should be robust in dealing with positional uncertainty, as objects such as flowers and leaves would seldom be completely stationary. Furthermore, the bee's flight is often perturbed by wind and turbulence. Nevertheless, in our experiments when the bees are within a few centimeters of the virtual objects, the spatial jitter will cause large angular deviations in the visual field. At this point the illusion may begin to break down, and the severe jitter may have a repulsive effect. This could explain why the final stage of descent toward a raised virtual background is markedly slower than that toward a static one (Figure 3C, blue vs. red dashed lines, *t* > 200 ms).

While the bees clearly avoided virtual obstacles, they did occasionally break through them (Figure 4) and eventually breached the raised background in the C60 test (Figure 3). Why did this happen? One possibility is that the aforementioned imperfections in the VR system eventually caused the illusion to break down and thus the bees ceased to perceive an object below them. Alternatively (or additionally), the animals could have been attending to ventrally-positioned visual cues other than the display (e.g., the ramp or monitor border), from which they could infer that they were in fact some distance clear of the floor. Indeed, given that the VR display is strictly limited to the ventral visual field, it is perhaps striking that the illusion is as effective as it is.

However, the aforementioned leg extension results (Figures 3D, 4D) indicate that the bees perceived the visual objects despite descending through them. It may be therefore that they were attempting to land on the objects, breaking through when their legs failed to make physical contact. Consistent with this account, they descended very slowly through the virtual background in C60 (Figure 3C). During such slow hovering flight, visual motion signals would become weak because of the lack of self-motion; bees may rely more on other sensory modalities (such as mechanosensory information from the outstretched legs) to guide the final moments of landing.

Yet another possibility is that because the target was untextured, it did not provide sufficient visual cues to gauge its proximity. As the bee drew near and the target occupied more and more of the ventral visual field, motion cues would have been eliminated. Essentially, they may have perceived the target as a circular gap in the obstacle through which a more distant blue background could be seen (see e.g., Supplementary Video 3). Closer inspection of the bees' flight maneuvers in the vicinity of the target could help to disentangle these possibilities: for instance, centered trajectories would suggest they saw it as a hole, whereas approaches to the edge would imply that they perceived it as a solid object (Lehrer and Srinivasan, 1993; Baird and Dacke, 2016). Unfortunately, the limited spatial resolution of our current setup—together with the infrequency of these collision events—makes it difficult to draw a conclusion either way. In any case, we do not feel that these occasional collisions with virtual objects seriously challenge the notion that visual motion plays a dominant role in flight control.

## Ethics statement

Animal experiments were performed in accordance with MEXT guidelines.

## Author contributions

EF and FS designed the experiment with input from NH. FS developed the VR system. EF performed the experiments. EF and FS analyzed the data. EF and FS wrote the manuscript. NH revised the manuscript.

### Conflict of interest statement

The authors declare that the research was conducted in the absence of any commercial or financial relationships that could be construed as a potential conflict of interest.

## Supplementary material

Supplementary Video S1 Beginning of a trial. Using the two sliding doors, a marked bee is selected and allowed to enter the experimental arena. The monitor is initially blank; closed-loop stimulus presentation begins when the bee first ascends to *z* > 350 mm. The condition shown in this instance is Htv (Figure 2, Supplementary Video 5).

Supplementary Video S2 C0 condition footage. Video recorded from the overhead camera in the no-VR control condition C0. Both the target and background appear at the monitor surface (z = 0 mm), so no closed-loop manipulation takes place. This mirrors the situation during training, except that no feeder is present. Leg extension is clearly visible shortly before the bee lands. Different individuals are shown in Supplementary Videos 2–9, except for Supplementary Videos 6, 9 which are the same bee.

Supplementary Video S3 C60 condition footage. Both the target and background are at a virtual altitude of 60 mm. The screen becomes blank pink when the bee descends below the virtual floor, as the target and background would no longer appear in the ventral visual field. Note that the bee hovers near the target edge, hesitating and extending its legs before descending through the virtual surface (Figure 3).

Supplementary Video S4 H condition footage. The bee flies through an 80 × 80 mm hole in the virtual surface (at *z* = 60 mm) to reach the target on the arena floor (*z* = 0 mm).

Supplementary Video S5 Htv condition footage. As H, but the target is visible at all times, even when it should be hidden from the bee's view by the foreground surface. The bee initially descends to an altitude close to the virtual foreground, but rather than approaching the target directly, ascends slightly and repositions in order to descend “through” the hole.

Supplementary Video S6 P condition footage. The bee flies around a virtual platform (at *z* = 60 mm) to reach the target on the arena floor (*z* = 0 mm).

Supplementary Video S7 Ptv condition footage. As P, but the target is visible at all times, even when it should be hidden from the bee's view by the platform. The bee initially approaches from a 5 o'clock direction, but it side-slips leftward and rotates right to land on the target from an 8 o'clock direction, thereby avoiding the virtual platform.

Supplementary Video S8 incHtv condition footage. A physically impossible situation where a “background” at *z* = 60 mm can be seen through a 120 × 120 mm hole in the “foreground” at *z* = 0 mm. Unlike the background, the target (which is also situated at *z* = 60 mm) is visible through the foreground. The target leaves the screen as the bee's altitude approaches 60 mm, because its position in the visual field drifts away from the nadir and toward the horizon. When the bee finally drops below 60 mm, the background disappears, revealing an untextured pink region.

Supplementary Video S9 incPtv condition footage. The same incongruent configuration as incHtv, but with a platform instead of a hole as the foreground object. The bee attempts to approach the target from inside the platform, contrary to the behavior observed in Ptv (Supplementary Video 7).
